# New Challenges in Wood and Wood-Based Materials

**DOI:** 10.3390/polym13152538

**Published:** 2021-07-31

**Authors:** Lubos Kristak, Ivan Kubovský, Roman Réh

**Affiliations:** Faculty of Wood Sciences and Technology, Technical University in Zvolen, T.G. Masaryka 24, 96001 Zvolen, Slovakia; kubovsky@tuzvo.sk (I.K.); roman.reh@tuzvo.sk (R.R.)

Wood and wood-based composites are key engineering materials that can be successfully designed and manufactured with predetermined exploitation properties, making them suitable for a wide range of applications and end uses. Notably, wood-based composites can be engineered to meet specific performance requirements, which makes them a sustainable solution for reducing the use of solid wood.

In the difficult times caused by the COVID-19 pandemic, we succeeded in compiling another interesting publication. It is a Special Issue of *Polymers* (ISSN 2073-4360) that belongs to the Section “*Biomacromolecules, Biobased and Biodegradable Polymers*” of MDPI. Even before the widespread outbreak of the pandemic, not knowing what was coming, the Special Issue was given the title “New Challenges in Wood and Wood-Based Materials”. Then the new challenges really came. We initially referred to different challenges. We certainly did not imagine that many researchers would have difficulty in routine scientific research work as a result of the significant disruption. The mobility of people has been restricted, access to workplaces has been more limited, and the preparation and compilation of scientific papers has become more complicated. Meeting the deadlines associated with the peer review process has been much more demanding than during normal times.

This Special Issue of *Polymers* is a collection of 11 original high-quality scientific contributions on basic and applied research in the field of wood science and technology, and provides good examples of the recent challenges related to the production and application of wood and wood-based materials. Individual papers concerned with the enhancement of the performance and technological properties of wood composites, above all plywood [1,2,3], as well as with ignition and combustion of wood and wood composites in monitoring and evaluating these processes on state-of-the-art equipment [4,5,6,7], and monitoring chemical changes in wood and wood adhesives and composites [8,9,10,11], are included. The topic of the Special Issue has clearly resonated in the world’s scientific community and the traditional response has come from strong wood research centers in Europe and Asia.

This Special Issue “*New Challenges in Wood and Wood-Based Materials*” follows up our previous Special Issue “*Application of Wood Composites*” in *Applied Sciences* [12]. In the first Special Issue, it turned out that wood and wood composite materials are engineered materials with significant physical, mechanical, or chemical properties which predetermine them for a whole range of uses, known or not yet discovered. Wood composites with their outstanding properties have replaced some conventional materials in various fields of applications. However, there is now a new and bolder goal: challenges. What are challenges? Something that by its nature or character serves as a call to make a special effort, a demand to explain or justify, or a difficulty in an undertaking that is stimulating to one engaged in it. Is this possible within the basic or applied research dealing with wood science and technology? The second Special Issue says quite clearly that we can also move within such boundaries of areas.

The scientific goal of this publication is to provide the reader with new information on recent practices in plywood research. Yes, plywood, as plywood is definitely back. Towards the end of the 20th century and also the beginning of the 21st century, there was a clear decline in plywood research. However, its unexpected increase in production and the expansion of its application also caused an increase in research activities. This is also reflected in this Special Issue. The study [1] investigated the effect of phenol-formaldehyde resin treatment on the weathering stability and biological durability of birch plywood. Silver birch veneers were vacuum-pressure-impregnated with four different phenol-formaldehyde resins with average molecular weights. The aging properties of phenol-formaldehyde resin-modified birch plywood were analyzed using artificial weathering with ultraviolet light, water spray, and weathering under outdoor conditions. The same combinations of phenol-formaldehyde-treated plywood specimens were then tested in soil-bed tests to determine their resistance against soft-rot wood decay. The weathering stability of birch plywood treated with phenol-formaldehyde resins has been proven. Results from unsterile soil-bed tests showed improvements in resistance to soft-rot wood decay compared to untreated plywood and solid wood.

A remarkable challenge is the utilization of large-scale wood in existing additive manufacturing techniques [2]. Wood-based materials in current additive manufacturing feedstocks are primarily restricted to the micron scale. This study proposes an additive manufacturing method—laser-cut veneer lamination—for wood-based product fabrication. Inspired by laminated manufacturing and common plywood technology, laser-cut veneer lamination bonds wood veneers in a layer-upon-layer manner. As demonstrated by printed samples, laser-cut veneer lamination was able to retain the advantageous qualities of additive manufacturing, specifically, the ability to manufacture products with complex geometries which would otherwise be impossible using subtractive manufacturing techniques. Furthermore, laser-cut veneer lamination product structures designed through adjusting internal voids and wood texture directionality could serve as material templates or matrices for functional wood-based materials. Numerical analyses established relations between the processing resolution of laser-cut veneer lamination and proportional veneer thickness (layer height).

The third work on plywood research deals with three dimensionally molded plywood formed parts [3]. They were prepared in two different geometries using cut-outs and relief cuts in the areas of the highest deformation. The effect of flax fiber reinforcement on the occurrence and position of cracks, delamination, maximum load capacity, and on the modulus of elasticity was studied. The results show that designs with cut-outs are to be preferred when molding complex geometries and that flax fiber reinforcement is a promising way of increasing the load capacity and stiffness of plywood formed parts.

Wood and wood-based materials can be subject to combustion, and therefore research into reducing their flammability requires long-term attention. This is also evident in this Special Issue. The study [4] focuses on the energy potential and combustion process of torrefied wood. Samples were prepared through the torrefaction of five types of wood: ash, beech, oak, pine, and spruce. They were heated under a nitrogen atmosphere. The samples enabled the investigation of torrefied wood combustion in a compact form. The effect of the external heat flux on the combustion of the samples was measured using a cone calorimeter. The observed parameters included initiation times, heat release rate, and combustion efficiency. The results show that increasing the external heat flux decreases the evenness of combustion of torrefied wood. At the same time, it increases the combustion efficiency. The results are useful both for the energy production field and for fire safety risk assessments of stored torrefied wood.

An experimental study of straw-based eco-panel using a small ignition initiator [5] points to another fundamental problem. It is well known that straw, a natural cellulose-based material, has become part of building elements and eco-panels, and compressed straw in a cardboard casing is used as building insulation because of its excellent insulating properties. If suitably fire-treated (insulation and covering), straw panels’ fire resistance may be increased. This study deals with monitoring the behavior of eco-panels exposed to a small ignition initiator (flame). The samples consisted of compressed straw boards coated with cardboard. Samples were exposed to a flame for 5 and 10 min. The influence of the selected factors (size of the board, orientation of flame to the sample) was compared on the basis of experimentally obtained data of mass loss. The results obtained do not show a statistically significant influence of the position of the sample and the initiating source (flame). The results presented in the paper confirm the justifiability of fire tests. As the results of the experiments prove, the position of a small burner igniting such material is also important. This weakness of the material can also be eliminated by design solutions in their construction. The experiment on larger samples also confirmed the justifiability of fire tests along with the need for flame retardancy of such a material for its safe application in construction.

If we talk about the protection of wood-based materials against fire, this area clearly includes oriented strand boards (OSBs). The experimental study of OSB ignition by radiant heat fluxes [6] investigated the ability of materials to ignite when heated at elevated temperatures. It depends on many factors such as the thermal properties of materials, the ignition temperature, critical heat flux, and the environment. OSBs without any surface treatment were used as experimental samples. The samples were gradually exposed to a heat flux. The ignition times are similar for all OSB thicknesses. The influence of the selected factors (thickness and distance from the heat source) was analyzed based on the experimentally obtained data of ignition time and weight loss. The results show a statistically significant effect of OSB thickness on ignition time.

The fire-technical properties of common woods such as spruce, oak, beech, etc. used in different constructions and buildings have been given much attention, while less attention has been paid to tropical woods as they represent more complex systems [13,14,15]. Selected tropical wood species (cumaru, garapa, ipe, kempas, merbau) were tested from the point of view of non-isothermal thermogravimetry [7]. For these tropical woods, a relationship was established between non-isothermal thermogravimetry runs and the wood weight loss under flame during cone calorimetry flammability testing. A correlation was found for the rate constants for decomposition of wood found from thermogravimetry and the total time of sample burning related to the initial mass. Non-isothermal thermogravimetry runs were assumed to be composed of three theoretical runs, such as decomposition of the wood into volatiles, oxidation of carbon residue, and the formation of ash. A fitting equation of three processes was proposed and the resulting theoretical lines match experimental lines. 

Other selected tropical woods (meranti, padauk) and merbau were tested from the point of the view of their changes in wood lignin during the ThermoWood process [8]. Thermal modification is an environmentally friendly process in which technological properties of wood are modified using thermal energy without adding chemicals, the result of which is a value-added product. Wood samples of three tropical wood species were thermally treated according to the ThermoWood process at various temperatures (160, 180, 210 °C) and changes in isolated lignin were evaluated by nitrobenzene oxidation (NBO), Fourier transform infrared spectroscopy (FTIR), and size exclusion chromatography (SEC). New data on the lignins of the investigated wood species were obtained, e.g., syringyl to guaiacyl ratio values. Higher temperatures cause a decrease in methoxyls and an increase in C=O groups. Simultaneous degradation and condensation reactions in lignin occur during thermal treatment, the latter prevailing at higher temperatures.

The chemical composition and morphological properties of Norway spruce wood and bark were evaluated in [9]. The extractive, cellulose, hemicellulose, and lignin contents were determined by wet chemistry methods. The dimensional characteristics of the fibers (length and width) were measured by Fiber Tester. The results of the chemical analysis of wood and bark show the differences between the trunk and top part, as well as in the different heights of the trunk and in the cross section of the trunk. The biggest changes were noticed between trunk bark and top bark. Fiber length and width depend on the part of the tree, while the average of these properties is larger depending on the height. Both wood and bark from the trunk contain a higher content of fine fibers and a lower content of longer fibers compared to the top. During storage, a decrease in extractives occurred, mainly in bark. Wood from the trunk retained very good durability in terms of chemical composition during the storage. In view of the morphological characteristics, a decrease in both the average fiber length and width in wood and bark occurred.

The growing need for sustainable products and the stringent legislative requirements related to the hazardous formaldehyde emissions from wood-based panels have boosted scientific and industrial interest in the production of eco-friendly, wood-based panels and optimal utilization of the available lignocellulosic materials [16,17,18,19,20,21]. The potential of the production of eco-friendly, formaldehyde-free, high-density fiberboard (HDF) panels from hardwood fibers bonded with urea-formaldehyde (UF) resin and a novel ammonium lignosulfonate (ALS) is investigated in the paper [10]. HDF panels were fabricated in the laboratory by applying a very low UF gluing factor (3%) and ALS content varying from 6% to 10% (based on the dry fibers). The physical and mechanical properties of the fiberboard, such as water absorption, thickness swelling, modulus of elasticity, bending strength, and internal bond strength, as well as formaldehyde content, were determined in accordance with the corresponding EU standards. The HDF panels exhibited very satisfactory physical and mechanical properties, fully complying with the standard requirements of HDF for use in load-bearing applications in humid conditions. Markedly, the formaldehyde content of the laboratory-fabricated panels was extremely low, ranging from 0.7–1.0 mg/100 g, i.e., meeting the most stringent requirements of the super E0 emission grade (≤1.5 mg/100 g), which allowed their classification as eco-friendly, low-emission, wood-based composites.

Last, but not least, the impact of wood waste on the mechanical and biological properties of silicone-based composites was investigated using wood waste from oak, hornbeam, beech, and spruce trees [11]. The density, abrasion resistance, resilience, hardness, and static tensile properties of the obtained wood–plastic composites were tested. The results revealed slight changes in the density, increased abrasion resistance, decreased resilience, increased hardness, and decreased strain at break and stress at break compared with untreated silicone. The samples also showed no cytotoxicity to normal human dermal fibroblasts. The possibility of using the prepared composites as materials to create structures on the seabed was also investigated by placing samples in a marine aquarium for one week and then observing sea algal growth.

We would like to thank to our section managing editor of the section “*Polymer Chemistry*”, Chris Chen, for all his assistance and ongoing support throughout the publishing process.

As the topic “*New Challenges in Wood and Wood-Based Materials*” is still relevant, i.e., there are emerging new challenges in wood and wood-based materials, it is understandable that MDPI has already opened submissions to a new Special Issue “*New Challenges in Wood and Wood-Based Materials II*” within the journal *Polymers* with the possibility of publishing work on a new wide range of wood and wood composite material challenges. We will be grateful for your further excellent scientific papers.
